# Multimodal sensory inputs and mechanosensory components mediate *C. elegans* negative gravitaxis

**DOI:** 10.1016/j.isci.2025.113923

**Published:** 2025-10-31

**Authors:** Caroline Ackley, Lindsey Washiashi, Neda Ziaei Kajbaf, Ruchira Krishnamurthy, Zhenxuan Sun, Vivian Duong, Kaylin Choe, Elijah Lane, Cricket Wood, Giulia Pellegrini, Eleanor Smith, Mark Sherwin, Pradeep Joshi, Joel H. Rothman

**Affiliations:** 1 Department of MCD Biology and Neuroscience Research Institute, University of California, California, Santa Barbara, CA 93106, USA; 2 Department of Neurology, University of California, California, San Francisco, CA 94158, USA; 3 University of Southern California School of Dentistry, Los Angeles, CA 90089, USA; 4 Cell and Developmental Biology, University of California, California, Los Angeles, CA 90095, USA; 5 College of Liberal Arts and Sciences, University of Illinois Urbana-Champaign, Urbana, IL 61801, USA; 6 Department of Physics, University of California, California, Santa Barbara, CA 93106, USA

**Keywords:** Behavioral neuroscience, Sensory neuroscience

## Abstract

The ability to sense Earth’s gravitational pull is essential for orientation, navigation, and proprioception in most organisms. We report here that *C. elegans* dauer larvae and adults show a pronounced tendency to move upward away from the center of the Earth. This behavior is antagonized by light and alternating electromagnetic fields, suggesting competition with multiple sensory modalities. Manipulating the direction and intensity of static magnetic fields does not reverse or eliminate this behavior, indicating that vertical migration is not a response to Earth’s geomagnetic field, but reflects negative gravitaxis. We screened known mechanosensory genes and found that MEC-5 collagen, MEC-7/12 tubulins, and TRPA-1, but not MEC-4/10 DEG/ENaC channels or other proteins involved in gentle touch transduction, are essential for gravitaxis. These findings implicate a mechanism for gravity sensation involving an ion channel that is also required for gravitaxis in flies, suggesting possible homology in gravity sensing across animal phylogeny.

## Highlights

• *C.elegans* negatively gravitax independent of geomagnetic fields
• Gravitaxis is attenuated by light and electromagnetic fields
• TRPA-1 and selected mechanosensory components are required for negative gravitaxis

## Graphical abstract

**Figure:**
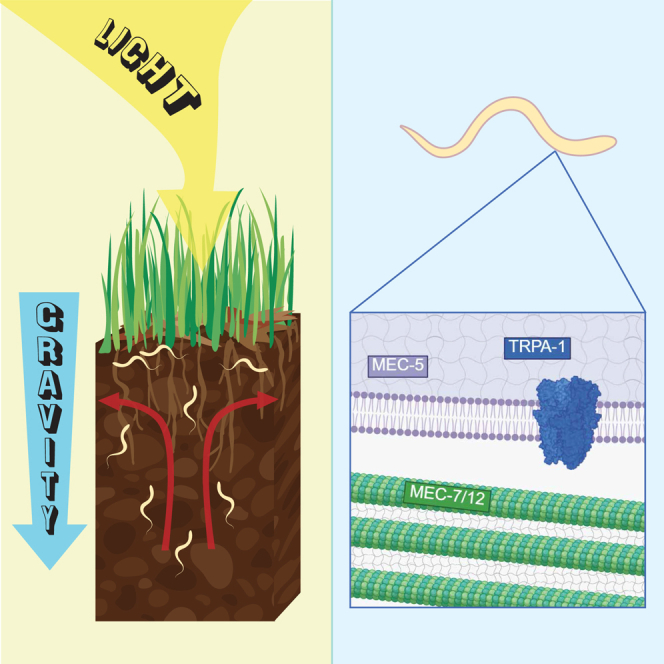

## Introduction

Gravity sensation is a trait common to most Eukaryotes. Members of the protists, fungi, plants, and animals depend on gravity sensation for survival. Small changes in the position or orientation of these organisms result in a mechanical force that is transduced by graviperceptive organelles or organs. This force is often conveyed through dense organelles or mineral-rich structures whose displacement triggers a signaling pathway that ultimately results in a behavioral output.^1^ A common characteristic of gravity transduction pathways in animals is the use of ciliated neurons, in which the deflection of hair-like “stereocilia” opens mechanically gated ion channels.^2^^,^^3^ Although this general mechanism has been well characterized, less is known about how mechanoreceptors and mechanosensory cells transduce such minute forces – estimated to be as small as 0.57–1.13 pN (pN) in single-celled *Euglena*, for example (which lack stereocilia) – into a robust signal.^4^

**Figure 1:**
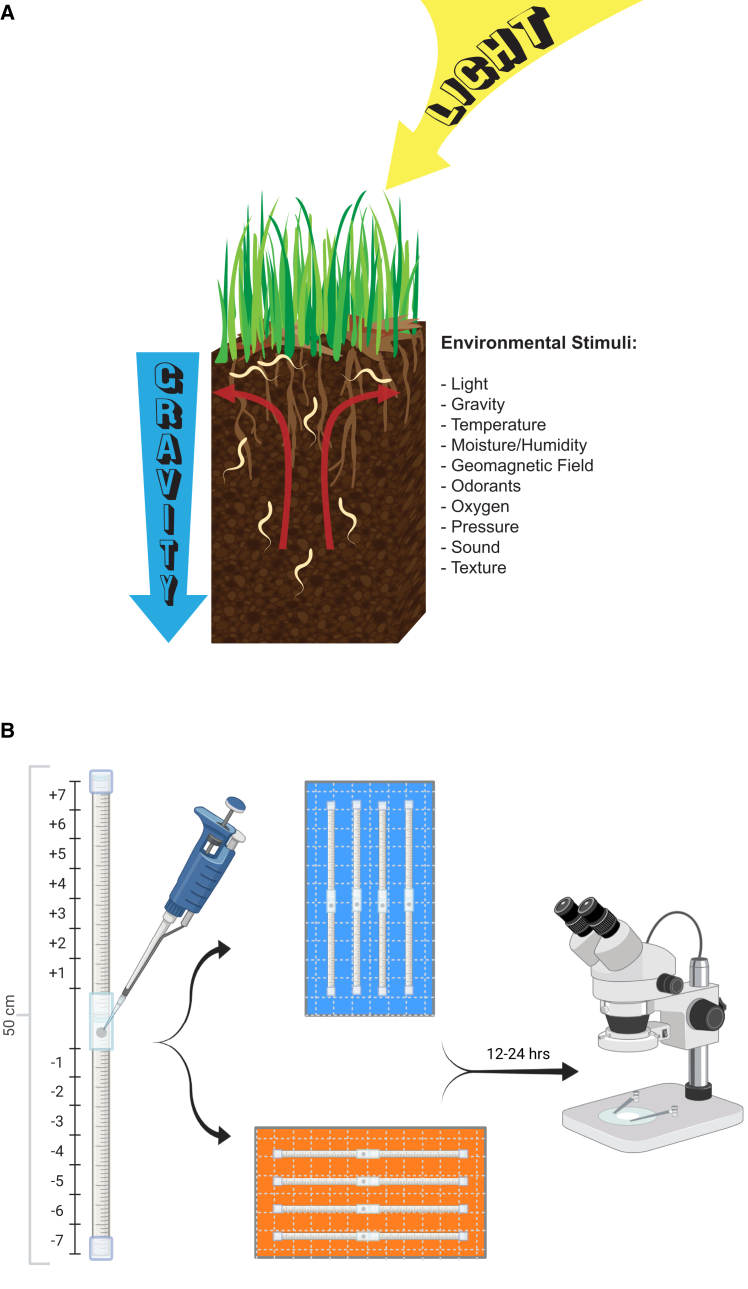
Behavioral model, wiring diagram, and gravitaxis assay design (A) Graphic depicts environmental stimuli that inform *C. elegans* behavior in a natural setting. (B) Gravitaxis chamber and experimental design used in this study. Figures (B) created in BioRender. Ackley, C. (2025) https://BioRender.com/ficosuu.

Given the availability of a detailed connectome of the entire nervous system and powerful genetic and optogenetic tools, *C. elegans* has been a highly effective model organism for elucidating the neural circuitry and molecular mechanisms governing sensory perception, learning, memory, and behavior. However, because they are traditionally studied in a two-dimensional environment on the surface of Petri dishes containing agar, perpendicular to the vector of gravity, few studies have examined the complex behavior exhibited by *C. elegans* in three dimensions.^5^ In the wild, *C. elegans* is typically found in moist compost, such as under shrubs or along riverbanks, which can change drastically from season to season.^6^^,^^7^ These animals have evolved an adaptive alternative larval phase, the dauer larvae, that prioritizes dispersal over reproduction during adverse environmental conditions. Dauer larvae exhibit characteristic nictation, in which they “stand” on their tails and wave their heads. This behavior is believed to facilitate dispersal, possibly by allowing worms to “hitch a ride” on larger animals that pass by, such as isopods.^8^^,^^9^ Therefore, gravitational force may be a critical input that *C. elegans* uses in combination with other cues to navigate to the surface before traveling longer distances (Figure 1A).

In a previous study, it was reported that *C. elegans* dauer larvae showed no gravitactic preference, in contrast with *C. japonica*, which negatively gravitaxes on vertically oriented Petri plates.^10^ Other studies reported that *C. elegans* adults show a tendency to orient downwards when swimming in liquid, suggesting potential positive gravitaxis.^11^ However, as in *Drosophila* and the ascidian *Ciona*, the ability of nematodes to undergo gravitaxis is likely context-dependent.^12^^,^^13^ *C. elegans* shows a strong aversive response to light^14^ and are highly responsive to electrical fields.^15^^,^^16^^,^^17^ It has been suggested that magnetotaxis can drive vertical taxis in *C. elegans*,^18^^,^^19^^,^^20^ although several other studies reported little or no responses to magnetic stimuli.^21^^,^^22^^,^^23^^,^^24^ Integration with these or other sensory modalities may influence or mask gravitactic behavior, and experiments to test for gravitaxis in the absence of light or electromagnetic fields (EMFs) have not been performed with *C. elegans*. In this study, we report that in the absence of light, *C. elegans* dauers exhibit pronounced negative gravitaxis, which is strongly enhanced when they are shielded from ambient electromagnetic fields and light.

While little is known about their behavioral responses to gravity, the effects of hyper- and microgravity on *C. elegans* physiology have been documented.^25^^,^^26^^,^^27^^,^^28^^,^^29^ In response to hypergravitational forces, signaling by the mechanosensory DEG/EnaC sodium channel proteins, MEC-4/MEC-10, leads to the nuclear localization of the DAF-16 FoxO transcription factor, which also transduces insulin-like growth factor signaling and stress responses.^30^ The *mec-4/10* genes in *C. elegans*, which encode channel proteins, and several other “*mec*” genes involved in gentle touch sensation, are expressed in the six touch receptive neurons (TRNs), which specifically mediate gentle touch sensation.^31^ Mechanosensation in many animals is also mediated by TRP (transient receptor potential) cation channels, which are common across metazoan phylogeny.^32^^,^^33^^,^^34^^,^^35^ The TRPA-1 receptor is a polymodal sensor, capable of conveying either high or low temperatures as well as light and noxious stimuli.^36^ In worms, TRPA-1 confers sensitivity to noxious cold in PVD neurons as well as mechanosensation in OLQ and IL1 neurons.^32^^,^^34^^,^^37^ The *trpa-1* homologs *pyx* and *pain* are necessary for gravitaxis in *Drosophila*.^38^

In this study, we sought to determine whether *C. elegans* possesses a system for detecting normal gravitational force and whether known mechanosensory molecular components and neurons participate in response to gravity. We discovered that *C. elegans* shows a pronounced tendency to migrate vertically against the force of gravity – i.e., to undergo negative gravitaxis. Further, we found that gravitaxis is profoundly influenced by environmental cues of light and background electromagnetic fields, revealing that competing sensory inputs modulate this behavior. Through several assays, we demonstrate that the direction of gravity, and not Earth’s magnetic field, is the primary influence on driving vertical taxis. We also report that the MEC-7/12 microtubule components and MEC-5 collagen protein, but not the associated MEC-4/10 DEG/EnaC channel proteins or several other components involved in TRN function and touch sensation, are required for gravitactic behavior. Finally, we show that the TRPA-1 channel is essential for negative gravitaxis in *C. elegans*. Based on these findings, we propose a model of gravity transduction that involves the unique action of MEC-5/7/12 and TRPA-1. Our findings suggest that even in a diminutive animal with low mass, a potentially homologous system for gravity sensing integrates with other sensory inputs to optimize responses to environmental cues and its orientation on the planet.

## Results

### *C. elegans* preferentially migrates vertically against the force of gravity

We investigated whether *C. elegans* can detect and respond to gravity by initially focusing on the behavior of the dispersal state, the dauer larva. When first stage (L1) larvae experience stressful conditions, including overcrowding, lack of resources, and extreme temperatures, they subsequently develop into an alternative third-stage larva, the dauer larva. Through pronounced physical, metabolic, and behavioral changes, dauers become efficient vectors for dispersal that can survive for months without food.^39^ Dauer larvae of many nematodes show nictation behavior, in which they raise their heads at a 90° angle to the surface plane, raising the possibility that they can orient in the gravitational field, though this behavior may simply reflect orientation perpendicular to the surface.

Under normal laboratory growth conditions in which *C. elegans* are cultivated on flat agar surfaces in Petri dishes, dauers of the laboratory N2 strain of *C. elegans* do not nictate unless contaminated by fungi or when grown on three-dimensional habitable scaffolds^5^; however, 3-D scaffolds are not convenient for studying migratory behavior. To address this issue, we adapted a setup used to study neuromuscular integrity^40^^,^^41^ and magnetotaxis^18^ in *C. elegans* to investigate whether *C. elegans* dauers exhibit directional bias in response to gravity^42^ (see STAR Methods). We injected dauers (approximately 500 microns in length) into the gravitaxis assay chambers comprised of two serological pipettes that, when stacked end-to-end, allow for ∼25 cm of movement in either direction from the injection site (Figure 1B). Chambers loaded with worms were oriented vertically to test for movement in the gravitational field, and migration of each individual was scored 12–24 h later. Positive gravitaxis (migration between bins −1 to −7) or negative gravitaxis (+1 to +7) was determined by comparing the distribution against paired normal distributions centered on the origin (see STAR Methods). In several experiments, we assayed horizontally oriented chambers simultaneously to control for any directional preference attributable to the construction and design of the chamber itself.

**Figure 2:**
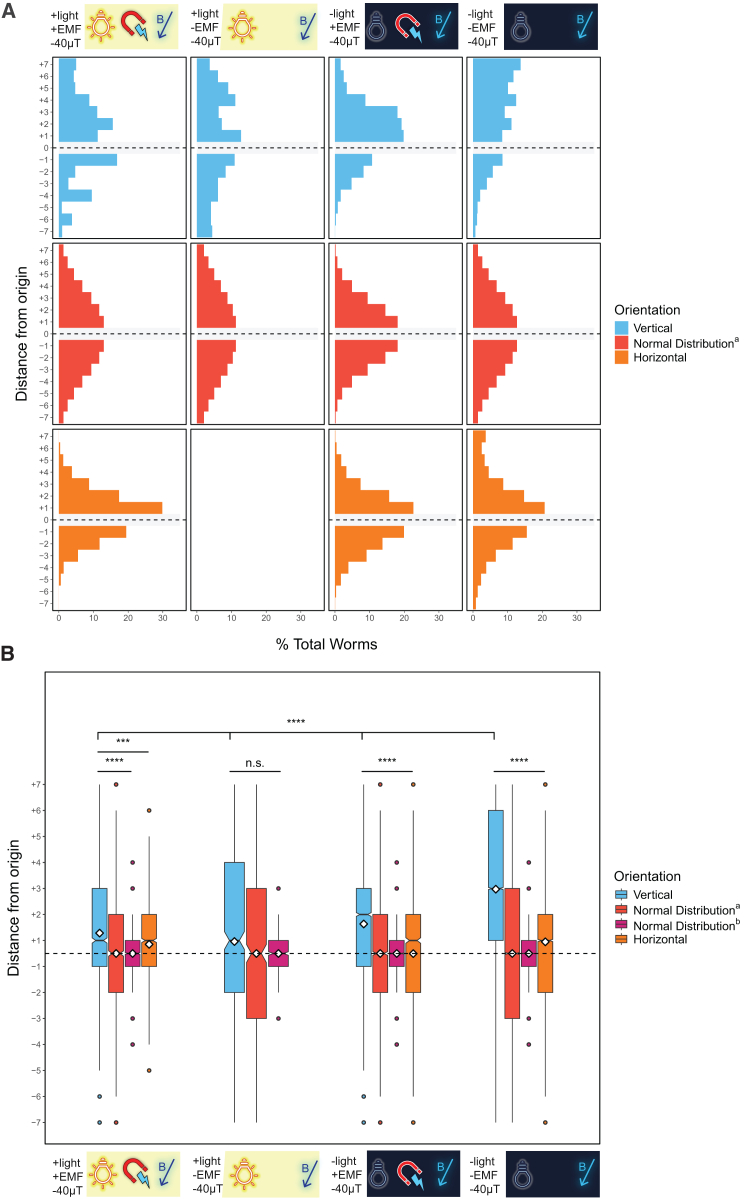
*C. elegans* undergo vertical migration under different environmental conditions (A) Histograms depict the cumulative distribution of *C. elegans* worms over multiple trials in vertical (blue) and horizontal (orange) assays as well as in comparison with a simulated normal distribution (Normal Distribution^a^), which is created using a mean of 0 and the same standard deviation as the combined vertical assay distributions. Horizontal gray box and dotted line indicate the center of the assay, which was not scored. Presence or absence of normal overhead light and background EMF (no Faraday cage) is denoted with +/−. −40 μT indicates normal exposure to Earth’s magnetic field, which is ∼ -40 μT in Santa Barbara, CA. (B) Boxplots depict the data shown in A. Dauers tested in a dark Faraday cage are used in future comparisons. ∗*p* < 0.05, ∗∗*p* < 0.01, ∗∗∗*p* < 0.001, ∗∗∗∗*p* < 0.0001; n.s. is not significant using Kruskal-Wallis followed by Dunn’s test with Bonferroni correction. Notches on boxplots represent 95% confidence intervals; mean values are indicated with a diamond. Normal distribution^a^ is described above; normal distribution^b^ was created using a mean of 0 and the same sample size as the combined vertical assays. Dotted horizontal line indicates the origin of the assay (location “0”), which was not scored. See also Figure S1.

In contrast to prior published assays conducted on Petri plates, which failed to reveal gravitactic preference with *C. elegans*,^10^ we found that N2 dauer larvae showed a weak but significant directional bias in migration toward the top of the vertical chamber under normal laboratory conditions compared to simulated normal distributions and horizontal controls (average vertical location = +1.28, as defined in STAR Methods, *n* = 2,222 worms over 10 trials; *p* < 0.0001, vertical vs. Normal Distribution^a^ and Normal Distribution^b^; *p* < 0.001, vertical vs. horizontal; Kruskal-Wallis test) (Figure 2A and 2B). For comparison, we applied a “Gravitaxis Index” (analogous to the Chemotaxis Index^41^^,^^42^) using the equation $GI=\frac{\left(Top-Bottom\right)}{total}$ and performed a two-sided *t* test against a hypothetical mean of 0. The results of this test were marginally significant, demonstrating the difficulty of assessing gravitaxis using a traditional behavioral index (GI vertical = 0.43 ± 0.17, SEM). Together, these observations suggest that *C. elegans* dauer larvae show a mild preference for upward migration under normal laboratory conditions when assayed across a migration field that is > 5x larger than that of previous studies.^10^

### Negative gravitaxis is attenuated by light and alternating electromagnetic fields

External and internal sensory cues that provide important context alter behavioral responses in animals.^43^^,^^44^ As *C. elegans* exhibits negative phototactic behavior,^14^ we posited that negative gravitaxis might be adversely influenced by this response to light (Figure 1A). To address this possibility, we repeated the gravitaxis assay in the dark using a light-blocking blackout cloth. In sharp contrast to the lack of discernible directional preference in the horizontal chambers, we found that N2 dauer larvae demonstrated an enhanced and highly significant preference for upward migration in the vertical chambers under these conditions (average vertical location = +1.63, *n* = 4,358 worms, 12 trials; *p* < 0.0001, all comparisons) (Figure 2A and 2B). This preference for upward migration was significantly greater (*p* < 0.0001) than that seen under normal laboratory lighting conditions (Figure 2B), suggesting that light attenuates the ability of the animals to sense or respond to the force of gravity.

*C. elegans* has also been reported to respond to direct^15^^,^^17^^,^^45^ and alternating^16^ currents, as well as magnetic fields.^18^^,^^19^^,^^20^ To test whether EMF, as with light, influences the gravitactic behavior of dauers, we analyzed their movement in chambers that were shielded from the ambient EMF present in the lab using a Faraday cage, which blocks EMF produced by alternating currents but not the magnetic field that is generated by the direct current of the Earth’s dynamo (see STAR Methods). We found that shielding the chambers from both EMF and light resulted in dramatically enhanced negative gravitactic behavior: in such chambers, dauers showed strong negative gravitaxis, with highly significant directional bias compared to horizontal controls and simulated normal distributions (average vertical location = +2.97, *n* = 34,766 worms, 142 trials; *p* < 0.0001, all comparisons) (Figures 2A and 2B). Further, the animals showed significantly stronger negative gravitaxis under these conditions than either the -light +EMF, +light -EMF, or +light +EMF conditions, when comparing the vertical distributions of all worms assayed (*p* < 0.0001 in all cases). Over the course of several months of use, we noticed that gravitaxis in our Faraday cage gradually decreased as a result of diminished EMF blocking, as confirmed by the restoration of cell phone reception in the cage (see STAR Methods). Strong negative gravitatic behavior was observed after reinforcing the cage (Figure S1). Thus, the ability of *C. elegans* to undergo negative gravitaxis is altered by both light and EMF, suggesting that gravitaxis behavior is integrated with other sensory inputs by the animal. In all subsequent experiments, we used a Faraday cage in the dark to block these other sensory inputs.

### Vertical movement is not in response to Earth’s magnetic field or temperature gradients

**Figure 3:**
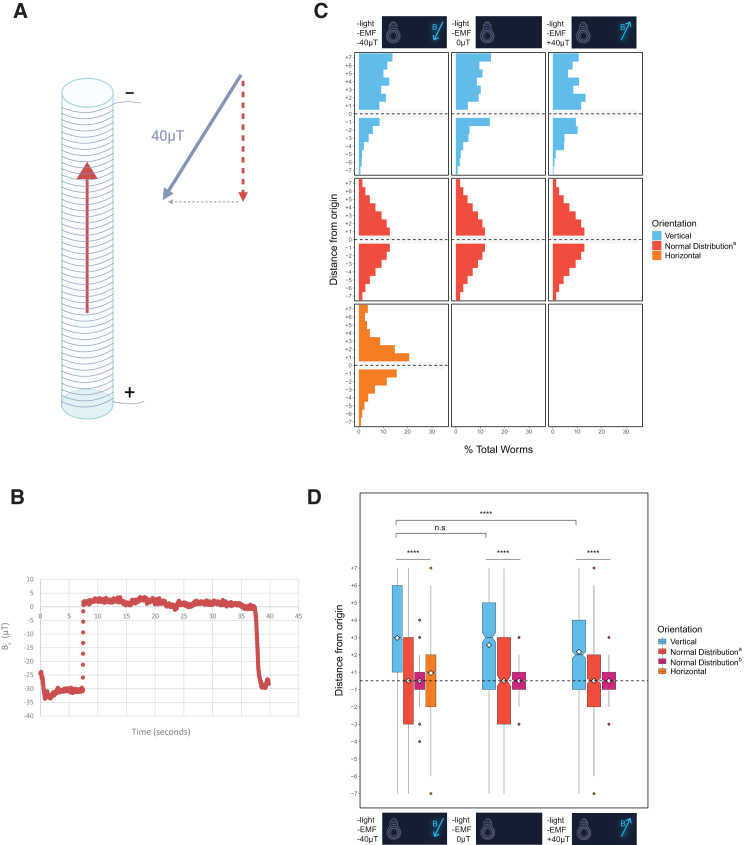
Vertical migration is not eliminated or reversed upon altering the magnetic field intensity or orientation (A) Diagram of the solenoid coil used to test *C. elegans* response to magnetic field perturbations in the gravitaxis assay. The strength and direction of the magnetic field in the laboratory were measured using the Physics Toolbox Suite app for iOS and Android (see STAR Methods). (B) The solenoid coil was used to counteract the vertical component of this field with measurements from a test run shown in (B) Created in BioRender. Ackley, C. (2025) https://BioRender.com/o0fkked. (C) Histograms depict taxis behavior under varying magnetic field conditions. Presence or absence of normal overhead light and background EMF (no Faraday cage) are denoted with +/−. −40 μT indicates normal exposure to Earth’s magnetic field, which is ∼ -40μT in Santa Barbara, CA. 0 μT and +40 μT conditions were assayed using a solenoid coil to manipulate the vertical component of Earth’s magnetic field in each experiment. (D) Boxplots depict data shown in A. ∗*p* < 0.05, ∗∗*p* < 0.01, ∗∗∗*p* < 0.001, ∗∗∗∗*p* < 0.0001; n.s. is not significant using Kruskal-Wallis followed by Dunn’s test with Bonferroni correction. Notches on boxplots represent 95% confidence intervals; mean values are indicated with a diamond.

Previous studies have debated the evidence for magnetosensation in *C. elegans*.^18^^,^^23^^,^^46^^,^^47^ While an early report claimed that the animals undergo magnetotaxis,^18^ subsequent studies have found no evidence that worms are magnetosensitive.^21^^,^^23^^,^^47^ In light of this controversy, we sought to determine whether the upward migration of the animals that we observed might be attributable to geomagnetism. As Faraday cages do not effectively shield against Earth’s magnetic field generated by direct current, we built a solenoid coil to mitigate the magnetic field strength along the vertical axis (B_z_) to determine whether magnetotaxis is responsible for upward movement. We assayed taxis behavior in a Faraday cage within the solenoid coil after reducing B_z_ from approximately −40 to 0 μT or reversing the magnetic field to +40 μT to assess whether worms were responding primarily to gravity or instead to vertically skewed static magnetic fields (Figures 3A and 3B; see STAR Methods). Under these conditions, we observed a strong upward migratory preference when the magnetic field was counteracted using a solenoid coil surrounding a Faraday cage (average vertical location = +2.56, *n* = 696 worms, 9 trials; *p* < 0.0001, all comparisons). In an even more convincing experiment, we found that fully reversing the magnetic field from −40 μT to +40 μT also did not substantially alter the tendency of the animals to move upward (average vertical location = +2.17, *n* = 1,112 worms, 9 trials; *p* < 0.0001, all comparisons, respectively) (Figure 3C and 3D). Thus, a static magnetic field cannot explain the tendency of the animals to migrate upward in the assay chambers.

**Figure 4:**
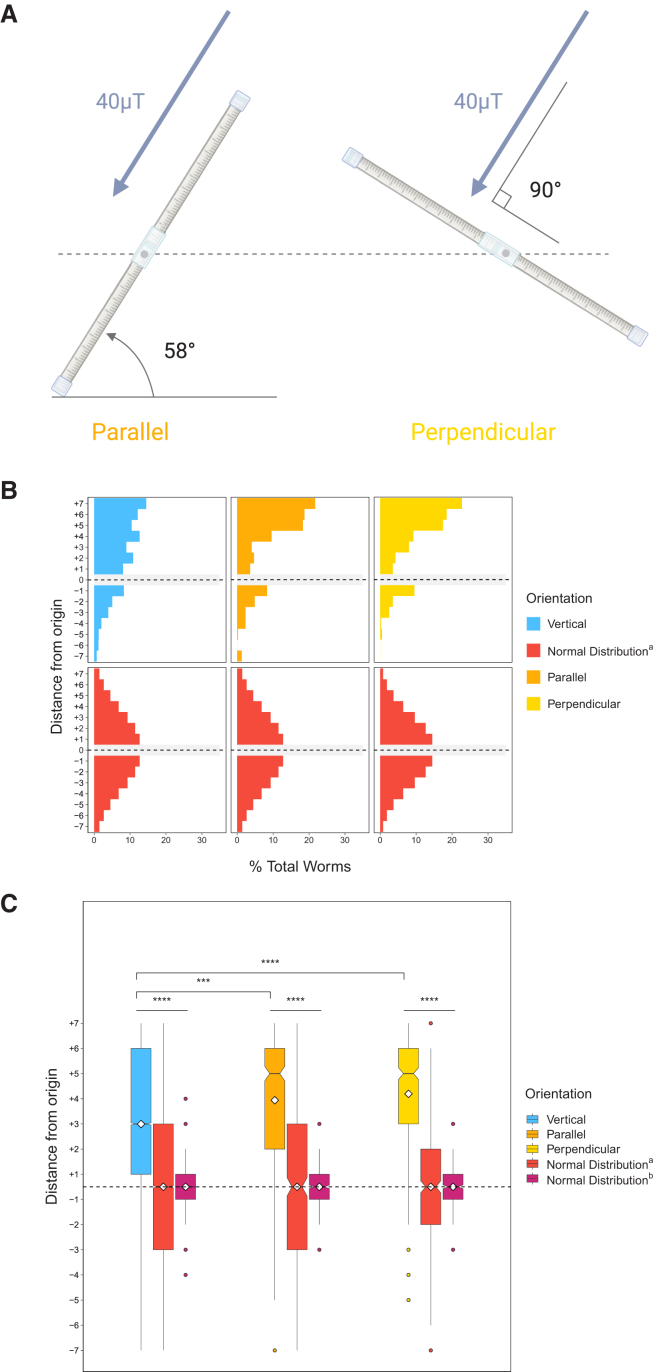
Worms oriented parallel or perpendicular to Earth’s magnetic field also exhibit vertical migration (A) Diagram represents the orientations of gravitaxis assays for this experiment. “Parallel” tubes were oriented at 58° from the ground, which was parallel to the local measured electromagnetic field (see STAR Methods for details). Conversely, “perpendicular” tubes were oriented 90° relative to the electromagnetic field. (B) Histograms depict vertical, parallel, and perpendicular movement compared with simulated normal distributions. (C) Boxplots summarize the data shown in A. Parallel *n* = 470 worms over 6 trials; perpendicular *n* = 400 worms over 5 trials. ∗*p* < 0.05, ∗∗*p* < 0.01, ∗∗∗*p* < 0.001, ∗∗∗∗*p* < 0.0001; n.s. is not significant using Kruskal-Wallis followed by Dunn’s test with Bonferroni correction. Notches on boxplots represent 95% confidence intervals; mean values are indicated with a diamond.

Supplementing the solenoid coil assay, we also performed experiments in which the chambers were rotated to align them either parallel or perpendicular to the vector of Earth’s magnetic field as it entered the Faraday cage. (The direction and strength of the geomagnetic field were determined using the Physics Toolbox Suite App for Android and iOS; see STAR Methods). If worms were primarily responding to Earth’s magnetic field, we would expect to see strong upward movement in the parallel, but not the perpendicular assay, as the effect of the field would be negated in the latter condition (Figure 4A). In both cases, however, we found a clear preference for upward migration (average vertical location = +3.94, *n* = 470 worms, 6 trials and +4.20, *n* = 400 worms, 5 trials, parallel and perpendicular assays, respectively; *p* < 0.0001, all comparisons) (Figures 4B and 4C). Thus, the orientation of the assay either along or perpendicular to the field was irrelevant to the movement of the animals away from the Earth’s center.

**Figure 5:**
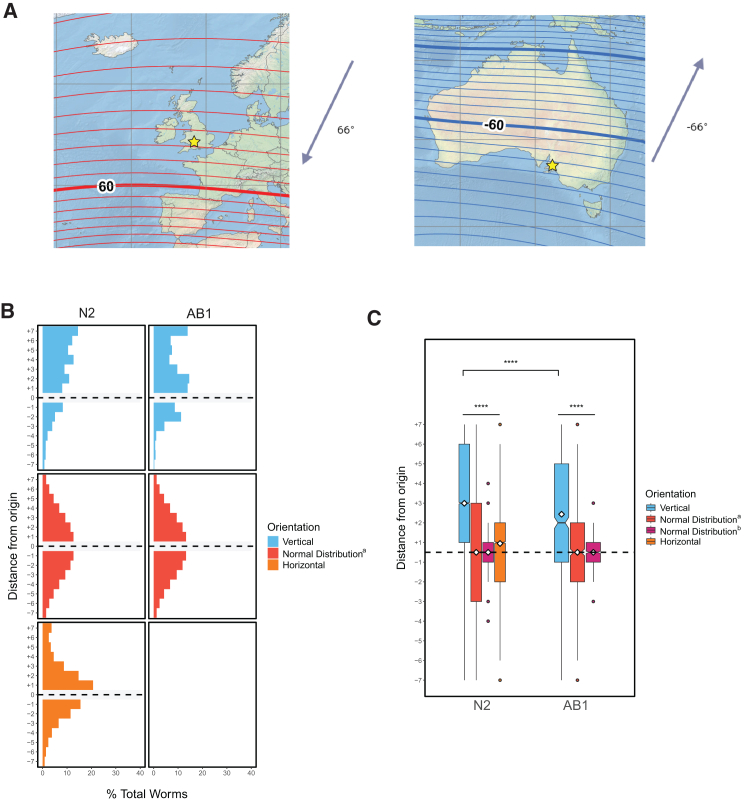
AB1 dauer larvae migrate vertically despite an inverted magnetic field inclination (A) Insets from the US/UK World Magnetic Model – Epoch 2020.0 Main Field Inclination (I) map developed by NOAA/NCEI and CIRES (available at https://ngdc.noaa.gov/geomag/WM). Red and blue lines indicate positive and negative magnetic field inclinations, respectively. Stars have been added to show Bristol, England, and Adelaide, Australia, where N2 and AB1 worms were each originally isolated. Created in BioRender. Ackley, C. (2025) https://BioRender.com/9nizdvi. (B) Histograms depict horizontal and vertical movement of N2 and AB1 dauers. (C) Boxplots summarize the data shown in A. AB1 vertical *n* = 775 worms over 8 trials. ∗*p* < 0.05, ∗∗*p* < 0.01, ∗∗∗*p* < 0.001, ∗∗∗∗*p* < 0.0001; n.s. is not significant using Kruskal-Wallis followed by Dunn’s test with Bonferroni correction. Notches on boxplots represent 95% confidence intervals; mean values are indicated with a diamond. See also Figure S2.

Evidence for magnetotaxis in *C. elegans* was previously supported by the observation that AB1 wild isolate worms, which are native to Adelaide, Australia, in the Southern Hemisphere, showed the opposite response to magnetic fields than N2, a strain isolated in Bristol, England^18^ in the Northern Hemisphere. As the inclination of Earth’s magnetic field in Bristol is oriented approximately 180° relative to the field direction in Adelaide (66°and −66°, respectively; Figure 5A), the investigators suggested that the opposing behavior of these strains could be explained by adaptation to their respective ambient electromagnetic fields. To further assess whether magnetosensation might guide vertical preference in our assay, we performed experiments comparing the movement of N2 and AB1 dauer larvae. We found that, similar to N2 dauers, AB1 dauers showed significant net upward migration compared with normally distributed data, albeit with a somewhat reduced bias (average vertical location = +2.44, *n* = 775 worms, 8 trials; *p* < 0.0001, all comparisons) (Figures 5B and 5C). While this difference in the strength of vertical preference may suggest other interesting variations between N2 and AB1 worm behavior, it contradicts the conclusion that upwards migration in this assay is primarily driven by magnetotaxis, as has been described previously.

Each of these findings complements results from other labs, which have not found behavioral responses to magnetic field direction or intensity,^21^^,^^22^^,^^23^^,^^47^ except in instances in which worms have ingested paramagnetic particles.^23^^,^^24^ Moreover, in examining the data from a prior published study that tested vertical migration behavior within a solenoid coil, we noted an unreported trend in vertical migration behavior in starved worms that is suggestive of gravitaxis behavior^47^ (Figure S2, data replotted in new format and reprinted with permission). While these data were collected over a shorter distance under various conditions and analyzed by the index method (counting the number of worms migrating up vs. down), comparison of the mean GI to a hypothetical mean of 0 revealed a statistical vertical preference in one instance when using a two-sided *t* test (GI = 0.17 ± 0.08, SEM; *p* < 0.05); however, it should be noted that this comparison was not found to be significant using the Wilcoxon signed-rank test, as was reported by the authors of that study. Together, these data collected across different laboratory settings point to a behavioral response that is consistent with gravitactic but not magnetotactic migration preference.

**Figure 6:**
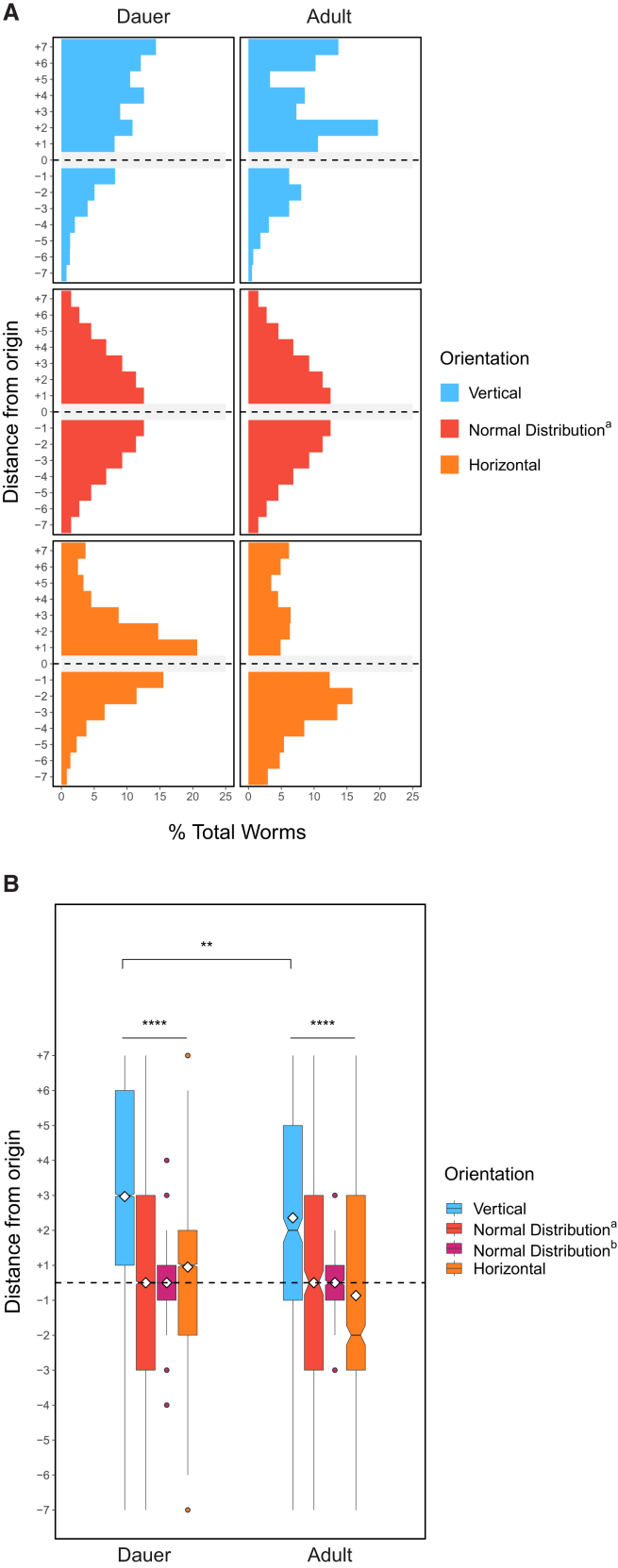
Adults negatively gravitax (A and B) Adult worms demonstrate a strong negative gravitaxis preference similar to dauers. ∗*p* < 0.05, ∗∗*p* < 0.01, ∗∗∗*p* < 0.001, ∗∗∗∗*p* < 0.0001; n.s. is not significant using Kruskal-Wallis followed by Dunn’s test with Bonferroni correction. Notches on boxplots represent 95% confidence intervals; mean values are indicated with a diamond. See also Figure S3.

Previous studies of magnetotaxis in *C. elegans* examined the behavior of adult worms under various feeding or starvation conditions.^18^^,^^23^^,^^46^^,^^47^ We considered the possibility that, similar to nictation,^8^ gravitaxis may be a behavior that is exclusive to dauers and not other developmental stages. Changes in gravitational response – or even in the ability to sense gravity – throughout development could also be important for the worms’ ecology, particularly given the importance of the dauer stage, but not adults, in dispersal of the animal. However, we found that, like dauer larvae, N2 adults also undergo significant vertical migration in the absence of light and EMF (average vertical location = +2.36, *n* = 548, 8 trials; *p* < 0.0001) (Figures 6A and 6B). While overall gravitactic preference in our vertical assay is reduced somewhat in adults compared with dauers (*p* < 0.01), we note that the distances traveled by adults and dauers may not be directly comparable, as adults are significantly larger and therefore travel shorter distances relative to their body length in this assay. Regardless, evidence of gravitactic behavior by adults suggests that gravity sensation may have a strong influence on behavior not only at the dispersal stage, but throughout *C. elegans* development.

One concern that may be raised when assaying worms in a solenoid coil or along a large vertical distance is that their behavior might be influenced by thermal gradients along the vertical axis. Indeed, when measuring the temperature at the top and bottom of our Faraday cage, we found a consistent difference of ∼1°C as a result of rising warm air in the laboratory. This difference corresponds to a gradient of <0.02 °C/cm, a differential that is substantially below the reported 0.1 °C/cm minimum threshold temperature gradient to which worms are known to be sensitive.^48^ However, as the reported threshold is limited by the accuracy of temperature measurements, it is conceivable that worms may be capable of detecting even shallower gradients. To clarify whether temperature preference might be responsible for the vertical migration, we reversed the temperature differential in our Faraday cage, such that the bottom of the cage was 1°C warmer than the top by providing a heat source at the bottom (see STAR Methods). Under this condition, we found that the pronounced upward migration persisted (average vertical location = +1.95, *n* = 445 worms, 5 trials; *p* < 0.0001, all comparisons), although the average vertical movement was somewhat lower than in the unaltered condition (*p* < 0.0001) (Figure S3).

Our findings indicate that the net vertical migration is not dramatically influenced by either magneto- or thermo-sensation. The pronounced upward migration of the animals is consistently maintained when the animals are shielded from light and alternating EMF, exposed to non-alternating magnetic fields in either of opposing directions, or subjected to weak temperature gradients in either orientation along the axis of the assay system. As there are no other obvious constant environmental influences that the animal is likely to experience that are oriented along the vertical axis besides the direction of Earth’s gravitational force under these various conditions, our findings provide strong support for the notion that it is the vector of the gravitational field that is responsible for the behavior, which we will henceforth refer to as negative gravitaxis.

### Elimination of the neural-expressed MEC-7/12 microtubule subunits and MEC-5 collagen, but not other components involved in gentle touch sensation, abolishes negative gravitaxis

Gravitational force is exceedingly weak compared with other mechanical stimuli, and particularly so for organisms with low mass. For a dauer worm of roughly 150 ng in mass, based on size and reported estimates of overall density,^49^^,^^50^ the Earth’s gravity would generate a maximum force of approximately 1.5 nN (see STAR Methods for calculations). By contrast, worms are sensitive to a gentle touch input of 1–10 μN.^51^^,^^52^ Diminutive organisms must therefore evolve extremely sensitive mechanisms for perceiving this minute force. In some small invertebrates, stretch receptors in the cuticle generate an ion influx upon deformation in a manner analogous to stretch and pressure sensors in mammals.^53^ The comprehensive set of molecular components that mediate gentle touch sensation in *C. elegans* has been identified through extensive genetic analysis.^52^ Because the gentle touch mechanosensory channel proteins MEC-4 and MEC-10 are also essential for transducing hypergravitational force in worms,^30^ we sought to determine whether these known mechanosensory components function in sensing Earth’s gravity.

**Figure 7:**
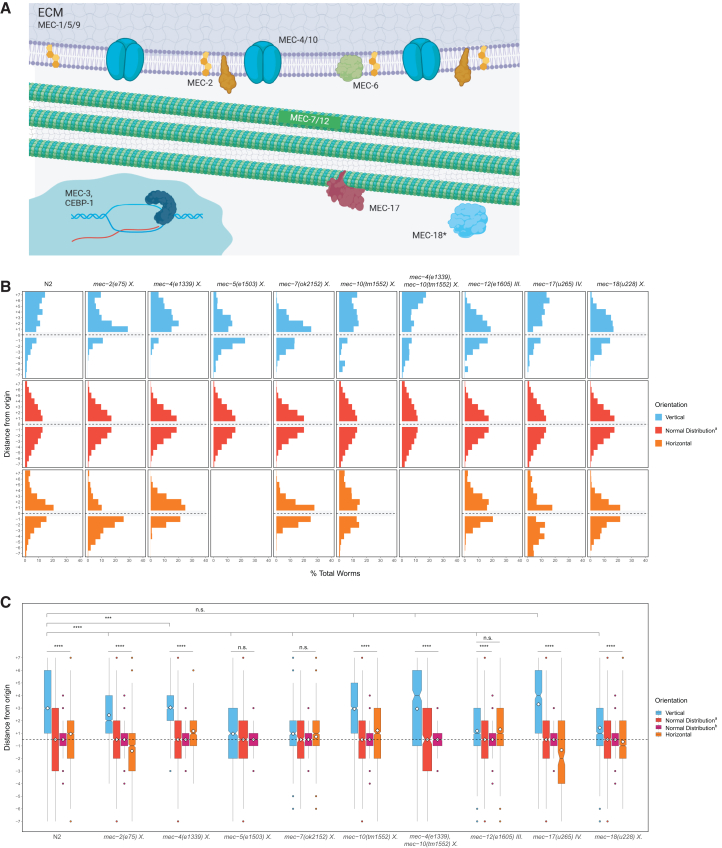
MEC-7/12 microtubule subunits, but not several other components in the gentle touch mechanosensory system, are necessary for gravitaxis (A) MEC-4 and MEC-10 subunits form a sodium channel that is required for gentle touch sensation in TRNs. MEC-1, -5, and -9 components of the extracellular matrix (ECM) form a structure between TRNs and the cuticle.^55^ MEC-7/12 microtubules form 15 protofilament structures in the TRNs only; however, these β and α subunits (respectively) are found in other neuronal 11 protofilament structures.^56^ MEC-2 proteins associate with both the Deg/EnaC channels and TRN microtubules and enhance channel activity, likely through interactions with cholesterol in the plasma membrane.^57^ MEC-17 is a transacetylase that stabilizes MEC-7/12 microtubules.^55^^,^^58^^,^^59^ MEC-18 (starred) performs a poorly understood role in the transduction of gentle touch. Based on its proposed function as an acetyl CoA ligase, we propose it may act in conjunction with MEC-17 to acetylate tubulin proteins. (Adapted from images by *Tavernarakis* et al. *1997* and others). Created in BioRender. Ackley, C. (2025) https://BioRender.com/v61h260. (B and C) Gravitaxis assays of N2 dauers and dauers carrying mutations in genes involved in mechanosensory transduction. (B) Histograms depict the cumulative distribution of *C. elegans* worms over multiple trials in vertical (blue) and horizontal (orange) assays as well as in comparison with a simulated normal distribution (Normal Distribution^a^), which is created using a mean of 0 and the same standard deviation as the combined vertical assay distributions. (C) Boxplots summarize data shown in A. ∗*p* < 0.05, ∗∗*p* < 0.01, ∗∗∗*p* < 0.001, ∗∗∗∗*p* < 0.0001; n.s. is not significant using Kruskal-Wallis followed by Dunn’s test with Bonferroni correction. Notches on boxplots represent 95% confidence intervals; mean values are indicated with a diamond. Normal Distribution^a^ is described above; Normal Distribution^b^ was created using a mean of 0 and the same sample size as the combined vertical assays. See also Figure S4.

We found that mutations that eliminated most components involved in the gentle touch response (Figure 7A) did not prevent gravitaxis in dauer larvae, demonstrating that gravity sensing is separable from touch reception. Gentle touch in *C. elegans* is perceived by the mechanosensory DEG/ENaC channels MEC-4 and MEC-10,^31^ which form a pressure-sensitive channel in the set of Touch Receptor Neurons (TRNs). We found that neither *mec-4(−)* nor *mec-10(−)* mutants were significantly defective for gravitaxis compared with wildtype controls (Figures 7B and 7C): *mec-4(e1339)* and *mec-10(tm1552)* mutant strains both exhibited a strong upwards directional bias in vertically oriented chambers compared to the horizontal controls and simulated normal distributions (average vertical location = +3.05, *n* = 1,340 worms, 7 trials and +2.94, *n* = 2,119 worms, 14 trials, respectively. *p* < 0.0001, all comparisons) (Figures 7B and 7C). An additional mutant strain of *mec-10* gave similar results (Figure S4). Analysis of *mec-4(−)* mutants is complicated by the observation that *mec-4*(*u253)* null mutant animals are sluggish^54^; this impaired movement could confound conclusions about the requirement for MEC-4 in gravitaxis. Indeed, when tested in our assay, *mec-4(u253)* worms showed severely defective movement and did not show clear gravitaxis (Figure S4). To rule out possible redundancy from either MEC-4 or MEC-10, we created and assayed a *mec-4(e1339); mec-10(tm1552)* double mutant strain, which similarly demonstrated a strong upward bias in our assay (average vertical location = +2.95, *n* = 481 worms, 6 trials; *p* < 0.0001, all comparisons). Together, our findings with the *mec-4*(*e1339*) missense mutant, which is touch-defective, and the *mec-10(tm1552)* null mutant suggest that the MEC-4/10 channel is not required for negative gravitaxis.

We observed that several other genes in the canonical gentle touch sensing system are similarly largely dispensable for gravitaxis. *mec-2* encodes a stomatin-like protein required for MEC-4/MEC-10 channel activity.^57^ MEC-2 binds cholesterol^60^ and likely facilitates gentle touch transduction by altering the composition of the plasma membrane surrounding MEC-4/MEC-10 ion channels.^57^ We found that *mec-2(e75)* dauers were not significantly impaired in their ability to gravitax when compared with the horizontal controls and normal distributions (average vertical location = +2.45, *n* = 2,621 worms, 10 trials; *p* < 0.0001, all comparisons) (Figures 7B and 7C). MEC-18 is required for gentle touch sensation, although its exact function has not been well characterized.^61^^,^^62^ *mec-18(u228)* mutants showed a strong gravitactic preference compared to controls (+1.44, *n* = 1,645 worms, 4 trials; *p* < 0.0001, all comparisons). While both mutants showed a significantly reduced vertical preference compared to N2 vertical distributions (*p* < 0.0001 in both cases), these experiments point to at most a minor role for these components in gravitaxis.

In contrast, disruption to MEC-5, a collagen protein associated with the gentle touch transduction pathway, resulted in a notable decrease in gravitactic behavior (average vertical location = +0.96, *n* = 1,051 worms, 6 trials; *p* > 0.05, all comparisons) (Figures 7B and 7C). Though research on MEC-5 in mechanosensation has largely focused on TRN/gentle touch transduction, MEC-5 is also expressed in ciliated head neurons^63^^,^^64^ and is essential for male mating behaviors through CEM neurons,^65^ suggesting a potential role for ciliated neurons in gravity transduction. We also found that a missense mutation in *mec-6* disrupted gravitactic behavior (average vertical location = +0.26, *n* = 195 worms, 6 trials; p ≫ 0.05, all comparisons). However, as with mec-4 null worms, these animals are known to be sluggish, consistent with their limited migration and narrow distribution around the origin of our assay (Figure S4).

The specialized MEC-12 alpha-tubulin and MEC-7 beta-tubulin proteins form unique 15-protofilament microtubules that are found only in the six TRNs and that are essential for mechanosensation. TRN microtubule bundles are thicker than the typical 11 protofilament microtubules found in most cells, including in other neurons.^66^ These 15 protofilament structures may provide intracellular resistance required for mechanotransduction; however, their exact function in this process is unknown.^56^^,^^67^ In contrast to our findings with other gentle touch sensation components, we found that removal of either MEC-7 or MEC-12 abolished negative gravitaxis, resulting in a random distribution in the chambers similar to that seen in the horizontal controls, although *mec-12* mutant strains differed significantly from simulated normal distributions (average vertical location = +0.97, *n* = 407, 4 trials; p ≫ 0.05, all comparisons for *mec-7(ok2152)* and average vertical location = +1.19, *n* = 5,099, 12 trials; p ≫ 0.05, horizontal comparison and *p* < 0.0001 comparing against normal distributions for *mec-12(e1605)*) (Figure 7B and 7C). A strain containing a missense mutation in *mec-7* similarly demonstrated little to no gravitactic preference (Figure S4).

MEC-7 and MEC-12 are required for normal axonal outgrowth^56^^,^^66^^,^^68^ and it was therefore conceivable that the *mec-7(−)* and *mec-12(−)* mutations might block gravitaxis primarily by altering neuronal development or structure. We were able to separate the role of MEC-7/12 in neuronal structure from its other functions by taking advantage of the *mec-12(e1605)* allele, an H192Y missense mutation that eliminates gentle touch sensation without detectably altering TRN development or structure.^69^^,^^70^ We found that gravitaxis is greatly diminished in *mec-12(e1605)* mutants (Figures 7B and 7C), suggesting that MEC-12 requirement in gravitaxis is separable from its role in neuronal morphology.

MEC-12 is the only *C. elegans* alpha-tubulin subunit known to be acetylated at K40.^70^^,^^71^ Mutations that modify K40 or that eliminate the MEC-17 transacetylase required for microtubule acetylation also prevent mechanosensation.^55^^,^^58^^,^^59^ We found that *mec-17(u265)* mutants, which lack functional MEC-17, undergo normal gravitaxis behavior (average vertical location = +3.31, *n* = 856 worms, 5 trials *p* < 0.0001, all comparisons) (Figures 7B and 7C) that did not significantly differ (p ≫ 0.05) from that of N2 animals, indicating that this modification, which is essential for stabilizing the MEC-7/12 microtubules in TRNs, is not required for sensation or response to gravity.

Taken together, these results support the notion that MEC-7/12 microtubules are essential for gravity perception in a role that is distinct from their structural roles or action in conferring gentle touch sensitivity.

### The TRPA-1 channel is essential for gravity sensation

Our finding that MEC-4 and MEC-10 are not required for gravitaxis suggests that other types of mechanosensory channels might instead participate in gravity perception. Prime candidates for such channels are members of the superfamily of TRP (transient receptor potential) proteins, which are implicated in many sensory modalities, including sensitivity to touch, hot and cold temperatures, noxious chemicals, and light.^32^^,^^36^ Orthologs of these channels have been found across metazoan phylogeny, including in all triploblast and diploblast animals, sponges, and even unicellular choanoflagellates, which are believed to be the closest surviving relatives of all metazoans.^72^ Notably, the TRPA-1 homologs *pain* and *pyx* are required for gravity sensation in *Drosophila*.^38^ TRPA1 is also expressed in the vestibular system, the primary organ where gravity sensation occurs in mammals.^73^ Though originally proposed as a putative mechanoreceptor in vestibular hair cells,^74^ studies in TRPA1 knockout mice have since shown no effect on balance or hair cell activity.^75^

**Figure 8:**
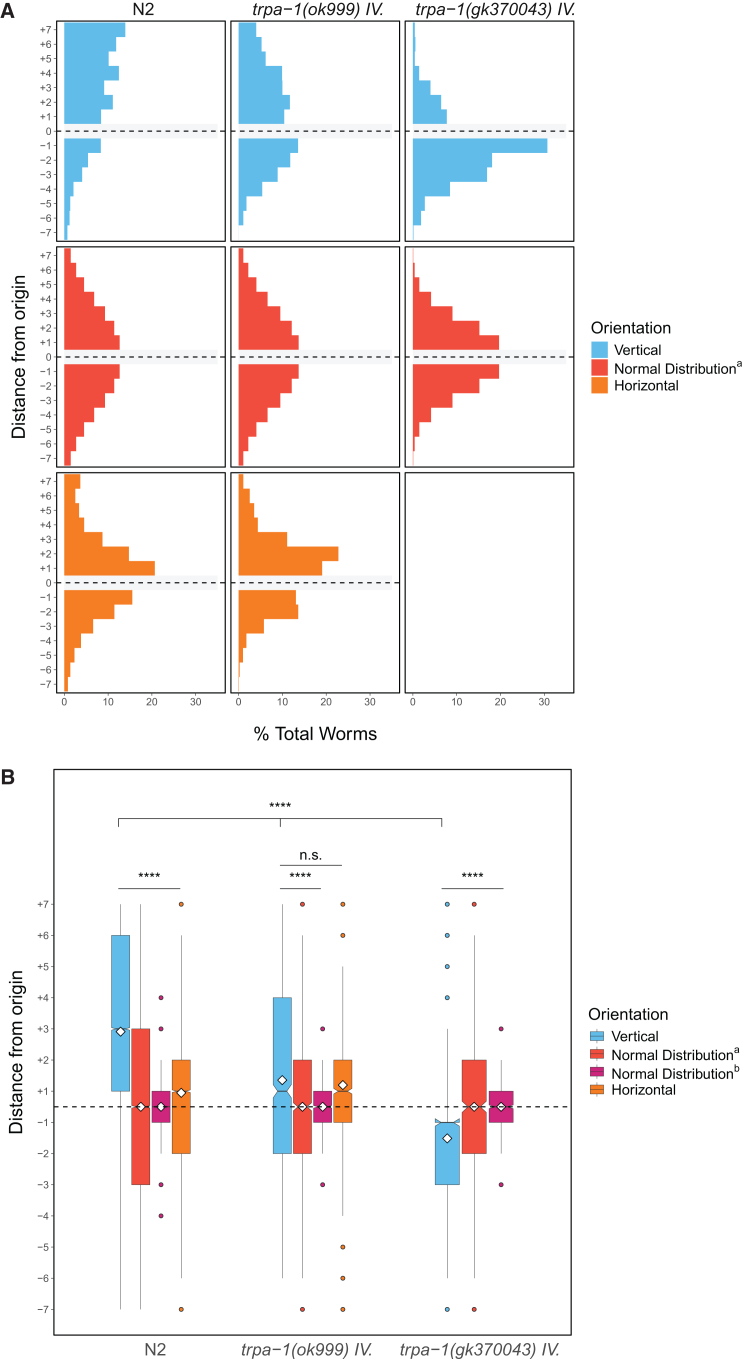
TRPA-1 channels are required for gravitaxis (A and B) *trpa-1* mutants in vertical (blue) and horizontal (assays) compared with N2 controls, as well as simulated normal distributions (described in previous figure legends and STAR Methods). ∗*p* < 0.05, ∗∗*p* < 0.01, ∗∗∗*p* < 0.001, ∗∗∗∗*p* < 0.0001; n.s. is not significant using Kruskal-Wallis followed by Dunn’s test with Bonferroni correction. Notches on boxplots represent 95% confidence intervals; mean values are indicated with a diamond.

We found that the reduced function of TRPA-1 in the *trpa-1(ok999)* mutant greatly reduced gravitaxis in dauer larvae (average vertical location = +1.36, *n* = 1,392, 10 trials; p ≫ 0.05 compared with a horizontal control, *p* < 0.0001, remaining comparisons) (Figures 8A and 8B). As *trpa-1(ok999)* mutant adult worms exhibit several movement defects, including decreased forward locomotion and several variations in the sinusoidal movement typical of wildtype worms,^76^ it was conceivable that the elimination of net upward biased movement of the animals might reflect diminished locomotory capacity rather than defects in gravity sensing *per se*. However, we found that *trpa-1(ok999)* dauer larvae distributed broadly across the chambers, comparable to that observed with N2 animals in both the horizontal and vertical conditions, demonstrating that these mutants are capable of traveling long distances regardless of orientation.

To further assess the requirement for TRPA-1 in gravitactic behavior, we assayed a second *trpa-1* mutant strain containing the *gk370043* variant. While *ok999* is a 1,334 bp deletion that removes most of exon 3, *gk370043* introduces a stop codon early in the gene, truncating the protein from 1,211 to 54 amino acids. Although both variants have a high variant effect predictor (VEP) impact, *gk370043* is likely to result in even more greatly reduced (i.e., abolished) protein function than *ok999*. In accordance with this prediction, we observed that *trpa-1(gk370043)* mutation showed an even stronger effect on negative gravitaxis than *trpa-1(ok999)* animals; in fact, these animals exhibit slightly *positive* gravitaxis behavior (average vertical location = −0.51, *n* = 697, 7 trials; p ≫ 0.05, all comparisons) (Figures 8A and 8B) raising the possibility that TRPA-1 might be required for the direction of orientation of migration along the gravitational field. These findings suggest that TRPA-1 channels are essential for, and may mediate, *C. elegans* gravitaxis.

### Limitations of the study

Our experiments, which eliminated all likely environmental factors other than gravity, including temperature and magnetic fields, support the conclusion that *C. elegans* is sensitive and responds to the vector of gravity. However, we note that this study is limited by our ability to manipulate the intensity and direction of the gravitational force experienced by worms using the Earth-bound assay. Studies of these effects would be advanced in microgravity aboard a spacecraft laboratory, which might provide additional insights into the threshold of force required for the behavioral response. We attempted to create a clinostat or centrifuge that would simulate micro- and hypergravity, respectively, but encountered challenges owing to the required physical length of our gravitaxis chambers, which generated a gradient of gravitational force in the device. These experiments were further complicated by the challenge of shielding these chambers completely from electromagnetic fields, which we have shown is essential to detect the behavior. Follow-up experiments should take these factors into consideration.

Additionally, while we identified several genes required for gravitaxis, further experiments will be required to elucidate their precise mechanisms in sensory transduction in this behavior. In this study, we have not explored the neural circuitry required for gravity sensation. An investigation of ciliated neurons and neurons expressing TRPA-1, MEC-7/12, and other components will further contribute to our understanding of gravity sensation in *C. elegans*. Because other competing sensory stimuli altered gravitaxis behavior in our assay, it would also be of interest to investigate crosstalk between sensory neurons and putative gravity-sensing cells.

## Discussion

In this study, we report four major advances. 1) We discovered that *C. elegans* dauer larvae and adults show pronounced negative gravitaxis. This drive toward movement away from the center of the earth may direct the animals toward food sources, typically decomposing vegetative matter at the surface.^6^ 2) We found that the ability of the animals to sense and respond to the force of gravity is attenuated by light and electromagnetic fields, suggesting that they integrate the sensation of these other environmental influences to make decisions about whether to undergo negative gravitaxis. 3) Vertical preference is not dramatically altered by manipulating static electromagnetic fields, implying that gravity and not Earth’s magnetic field is primarily responsible for this behavior. 4) The ability of the animals to sense and/or respond to gravity does not require DEG/ENaC channels or other factors involved in touch sensation but does require the MEC-7/12 microtubule components, MEC-5 collagen, and TRPA-1 channels. Thus, gravity sensing involves a previously unknown system involving specialized microtubules, which also participate in touch sensation and may function with TRP sensory channels.

Our findings that *C. elegans* dauer larvae exhibit pronounced negative gravitaxis contrast with prior studies, which reported no gravitactic behavior in dauers, or that in which positive reorientation toward the gravitational vector in adults in a liquid suspension was observed.^10^^,^^11^ These differences are likely attributable to differences in several parameters of assay design. Previous experiments have been performed on Petri dishes, which limit the distance animals can travel. Owing to the long distances traversed in our chambers, we were able to find robust and highly significant differences between vertically oriented chambers and normal distributions or paired horizontal controls. In addition, the environment of a worm crawling in the thin space between the agar substrate and the wall of the chamber may be more similar to the act of crawling through a column of soil than the experience of moving along the agar surface at the interface with the atmosphere on a plate. Finally, earlier experiments on gravitaxis in *C. elegans* made no mention of the lighting conditions used or shielding against EMF, leading us to propose that competing sensory inputs, including light and alternating magnetic fields, may have attenuated the behavior in those studies, as we have demonstrated here.

Underground habitats are generally ill-suited for *C. elegans*. Such environments may cause dauer larvae to find their way to the surface, where decaying vegetation might be present. However, the animals also avoid light,^14^ which is associated with damaging ultraviolet radiation. While studies suggest that the animals may navigate using the Earth’s magnetic field,^18^^,^^19^^,^^20^ subsequent studies have not replicated this finding.^21^^,^^22^^,^^23^^,^^47^ Our finding that ambient light and EMF in the laboratory setting strongly attenuate gravitactic behavior – likely explaining why negative gravitaxis had not been reported previously – suggests that the animals compare sensory inputs from a variety of environmental signals, including the force of gravity, to optimize behavioral decisions^77^ in complex environments. Additionally, by observing gravitaxis behavior under conditions in which the weak effects of Earth’s magnetic field and shallow thermal gradients are further minimized, we found that gravity, and not magnetic or temperature stimuli, is the only obvious influence that leads to upward migration. However, we also note that, while reversing or reducing the intensity of static electromagnetic fields did not eliminate the behavior, it did reduce overall gravitaxis compared to assays performed in −40 μT. The extent to which light, EM fields, and potentially other inputs override the response to gravity, as well as other sensory neurons and the circuits required to compute their respective influence on behavior, are important outstanding questions.

Although it was not detected in earlier studies, our altered assay system, which effectively shields the animals from other confounding environmental stimuli, revealed that both dauers and adults of *C. elegans* exhibit pronounced negative gravitaxis. The reduced ability of N2 dauers to undergo negative gravitaxis compared to that observed with *C. japonica* dauers might be an outcome of myriad adaptations to laboratory cultivation, as has been observed for several other traits, including social feeding, egg laying behavior, oxygen tolerance, and nictation.^8^^,^^78^^,^^79^^,^^80^ We found that negative gravitactic behavior, albeit weakened, is also seen with the LSJ1 sister strain (Figure S4), which has been raised in liquid media over many decades, as well as in *C. briggsae*.^42^ It remains an open question as to whether these strains show negative gravitaxis when shielded from light and EMF when swimming in liquid rather than crawling on solid media.

Our finding that TRPA-1*,* MEC-5 collagen, and MEC-7/12 microtubule functions are required for gravitaxis reveals an unexplored mechanism for transducing gravitational force. Unlike the DEG/ENaC channels, TRPA-1 receptors do not require internal or external machinery for their function.^34^ Additionally, *trpa-1* is expressed in only one of the six TRNs, which are the only neurons containing the 15 protofilament structures formed by MEC-7/MEC-12 tubulins. MEC-7/MEC-12 microtubules may perform more than a structural role in mechanosensation; whether this function is independent of their assembly into 15 protofilament structures has yet to be determined.

The identification of these genes as essential for gravitaxis hints at a novel mechanosensory pathway in *C. elegans*. Although TRPA-1 is critical for gravitaxis in our assay, additional studies are necessary to determine its precise role in gravity transduction. TRPA-1 and orthologous genes are known to act as both primary and secondary sensory receptors in a variety of pathways.^35^ Therefore, TRPA-1 may act downstream of other mechanoreceptors to facilitate gravity sensation. Likewise, the combination of TRPA-1 and MEC-5/7/12, but *not* MEC-4/10, as critical components provides insight into which neurons may be required for gravitaxis in *C. elegans*. MEC-5, -7, and -12, are each expressed in several neurons^63^^,^^64^ and other cell types throughout the worm, but their function beyond gentle touch sensation in TRNs has not been fully explored.

An examination of which cells express *trpa-1* and *mec-5*, *-7*, and *-12,* and knowledge of gravity perception in other species can also narrow a list of potential gravity-sensing neurons that might mediate this behavior. It is noteworthy that ciliated neurons are frequently used in gravity sensation, including in humans.^2^^,^^3^ *C. elegans* contains 60 ciliated neurons,^73^ 38 of which express both *mec-12* and *trpa-1*^61^^,^^62^ and some of which may require MEC-5 collagen in order to function properly.^63^ Of these 38 cells or cell pairs, 5 (AFD, ASH, AQR, OLQ, and PHA) also express an orthologue of the likely gravity-sensitive mechanoreceptor in mammals, TMC-1.^63^^,^^64^^,^^81^^,^^82^^,^^83^ AFD neurons are of particular interest because they were identified as mediating magnetotactic behavior.^18^^,^^19^ Neurons involved in proprioception, including FLP neurons located in the head and PVD neurons, which span the entire length of the worm, are also candidates.

Understanding how *C. elegans* perceives gravity has implications for both invertebrate and mammalian biology. *C. elegans* has been used for decades as a model for understanding the effects of microgravity on animal physiology in space,^25^^,^^26^^,^^29^^,^^84^^,^^85^^,^^86^^,^^87^^,^^88^ and yet scant information is available regarding their ability to sense gravity. Moreover, gravity perception is linked evolutionarily and developmentally with hearing^89^^,^^90^: the vestibular and auditory systems in mammals share similar sensory structures, transduction machinery, and gene expression.^91^ A role for TRPA1 has been identified in sensory transduction within inner ear hair cells in mice.^74^ The similarities between these systems make the study of gravity sensation important not only for understanding mammalian vestibular sensation, but also for auditory sensation. Thus, uncovering the role of TRPA-1 as a mechanoreceptor raises the possibility that *C. elegans* could be used as a model for studying both vestibular sensation and audition. The warping of space-time caused by Earth’s mass is among the most consistent stimuli experienced by organisms across billions of years of evolution. Our results raise the exciting possibility that a mechanism for detecting this stimulus may have been preserved since at least the divergence of the nematode (*C. elegans*) and chordate (e.g., mammals) phyla over 500 million years ago.

## Resource availability

### Lead contact

Further information and requests for resources and reagents should be directed to and will be fulfilled by Joel Rothman (rothman@lifesci.ucsb.edu).

### Materials availability

Worm strains generated in this study will be made available upon request.

### Data and code availability

• All data reported in this article will be shared by the lead contact upon request.
• This article does not report original code; scripts created for data analysis and visualization will be made available upon request.
• Any additional information required to reanalyze the data reported in this article is available from the lead contact upon request.

## Acknowledgments

Funding for this work was provided in part by 10.13039/100000002NIH grants #R01GM143771 and R01HD081266 to JHR. CA was supported in part by fellowships from the Department of Molecular, Cellular, and Developmental Biology as well as the President’s Dissertation Year Fellowship through UCSB Graduate Division and an American Rescue Plan (ARP) Grant through HEERF III. LW was funded as a Dr. Rajendra Singh Fellow through the UCSB College of Creative Studies. NZK received support through the Gorman Scholarship Program at UCSB. RK received funding as a UCSB Academic Research Consortium Scholar. Sequencing was performed at the DNA Sequencing Facility at UC Berkeley. Statistical consultation was provided by the DATALAB in the UCSB Department of Statistics and Applied Probability. Some strains were provided by the Caenorhabditis Genetics Center, which is funded by the NIH Office of Research Infrastructure Programs (P40 OD010440). We are grateful for the generous gifts of transgenic worms provided to us by the Chalfie Lab (Columbia University).

## Author contributions

CA: conceptualization, methodology, formal analysis, investigation, writing – original draft, visualization, and supervision. LW: methodology, validation, and investigation. NZK: methodology, validation, and investigation. RK: investigation and formal analysis. ZS: methodology, validation, and investigation. VD: methodology, validation, and investigation. KC: methodology, validation, and investigation. EL: methodology, validation, and investigation. CW: conceptualization and methodology. GP: methodology and investigation. ES: methodology, validation, and investigation. MS: project administration. PJ: conceptualization and writing – review and editing. JR: conceptualization, writing – review and editing, project administration, and funding acquisition.

## Declaration of interests

The authors declare no competing interests.

## STAR★Methods

### Key resources table

**Table:** 

REAGENT or RESOURCE		SOURCE	IDENTIFIER
**Experimental models: *C. elegans* strains**			

N2	Wild isolate	Caenorhabditis Genome Center (CGC)	WBStrain00000001
AB1	Wild isolate	Caenorhabditis Genome Center (CGC)	WBStrain00000032
LSJ1	Wild isolate	Caenorhabditis Genome Center (CGC)	WBStrain00026322
CB75	*mec-2(e75) X.*	Caenorhabditis Genome Center (CGC)	WBStrain00004092
CB1339	*mec-4(e1339) X.*	Caenorhabditis Genome Center (CGC)	WBStrain00004303
CB1611	*mec-4(e1611) X.*	Caenorhabditis Genome Center (CGC)	WBStrain00004360
TU253	*mec-4(u253) X.*	Caenorhabditis Genome Center (CGC)	WBStrain00035037
JR4521	*mec-4(e1339) X.;* *mec-10(tm1552) X.*	This paper	N/A
JR4614	*trpa-1(gk370043) IV.* (outcrossed x2)	This paper	N/A
CB1503	*mec-5(e1503) X.*	Caenorhabditis Genome Center (CGC)	WBStrain00004349
CB1472	*mec-6(e1342) I.*	Caenorhabditis Genome Center (CGC)	WBStrain00004340
RB1708	*mec-7(ok2152) X.*	Caenorhabditis Genome Center (CGC)	WBStrain00032400
CB1477	*mec-7(e1343) X.*	Caenorhabditis Genome Center (CGC)	WBStrain00004341
ZB2551	*mec-10(tm1552) X.*	Caenorhabditis Genome Center (CGC)	WBStrain00040801
CB1515	*mec-10(e1515) X.*	Caenorhabditis Genome Center (CGC)	WBStrain00004352
CB3284	*mec-12(e1605) III.*	Caenorhabditis Genome Center (CGC)	WBStrain00004467
TU265	*mec-17(u265) IV.*	Caenorhabditis Genome Center (CGC)	WBStrain00035038
TU228	*mec-18(u228) X.*	Caenorhabditis Genome Center (CGC)	WBStrain00035036
TQ233	*trpa-1(ok999) IV.*	Caenorhabditis Genome Center (CGC)	WBStrain00034933

**Oligonucleotides**			

*mec-4*	248 bp	F: TTTGACATGCAACACTCACCA	R: GATCACGAAGAGGAACGCGA
*mec-10*	1592 bp	F: CGTAGTCGCAGTCGATTTCA	R: ATCGGAAAACCAACACTTGC

### Experimental model and subject details

*C. elegans* worms were grown and maintained on OP50 *E.coli* at room temperature (23°C) using standard methods.^92^ Dauer formation was induced by overcrowding, which occurred 8–12 days after chunking onto seeded NGM plates. Adults used for gravitaxis assays and imaging were taken from synchronized populations 2 days after hatching. Crosses were performed as described previously,^93^ using fluorescence microscopy, PCR, and/or sequencing to confirm the outcome of each cross.

All strains used in this study are cataloged in the supplemental information.

### Method details

#### Dauer isolation

Dauer larvae were isolated from 1 to 5 starved NGM plates using 1% SDS and a 30% sucrose gradient as described previously.^94^^,^^95^ Briefly, worms were rinsed and collected in M9, then pelleted and resuspended in 7 mL 1% SDS in a 15 mL falcon tube. Worms were left rotating for 30 min in SDS to kill all non-dauer life stages. After 3–5 rinses with M9, worms were resuspended in 10 mL 30% sucrose solution and centrifuged for 5 min. Surviving dauers were collected at the interface of the water-sucrose gradient with a large bore glass Pasteur pipette and rinsed 3-5X in M9. Dauers were used immediately or after rotating in solution overnight.

#### Synchronization

Mixed stage worms were rinsed from confluent plates with M9 and collected in 1.5 mL tubes. Worms were then rinsed and pelleted. A 1 mL bleach solution containing 150 μL bleach and 50 μL 5N KOH was added and worms were lysed in this solution for 3 min, or until most corpses were dissolved. The embryo pellet was then rinsed 3-5X in M9 and plated onto OP50 NGM plates until adulthood (∼2 days at 23°C).

#### Assay preparation

Gravitaxis assay chambers were created using 5 mL serological pipettes and 4% NGM agar as described previously.^42^ To assemble each chamber, a heated blade was used to cut the tapered end of one pipette and the cotton plug was removed from the end of a second pipette. The two pipettes were then joined end-to-end by melting the two ends slightly over a Bunsen burner and fusing them with moderate pressure, ensuring that no gaps remained. The NGM media was then autoclaved and taken up by each double pipette using a standard serological pipettor while the agar was still molten. Best results were achieved by orienting each pipette parallel with the benchtop as much as possible during the procedure and as the agar solidified. Pipettes were used within 24 h of construction or stored at 4°C to be used within 48 h.

Once solidified, a heated 3 mm Allen key was used to punch a hole through one wall of the pipette about 5 mm below the fusion point (for reference, the cotton-plug end is the “top”). Each end of the pipette was removed using a heated blade and sealed with parafilm.

#### Gravitaxis assay

Worms were pelleted in M9 and were either extracted directly from the pellet or pipetted onto a square of parafilm. After cutting the tip to increase the bore, a pipette was used to aspirate 0.5–2 μL of the concentrated worm pellet, letting in a small volume of air at the end to facilitate injection. Worms were injected into the agar at the opening created by the Allen key. This opening was then sealed with parafilm, and the chamber was immediately oriented vertically or horizontally. Each chamber represents an experimental trial in which worms were later scored individually. Separate conditions (such as different strains or environmental variables) were always run in parallel with at least one (but often multiple) N2/vertical/Faraday cage control.

Unless indicated otherwise, all experiments were performed in a custom-manufactured Faraday cage. The effectiveness of the cage in blocking EMF was tested by placing a cell phone within the cage and calling it to determine if it received a signal. Worms were allowed to gravitax overnight and were scored the following day (typically 12–24 h after injection).

Pipettes were examined under a dissecting microscope and a marker was used to mark the location of each worm. Pipettes were scored immediately after removal from the assay location. Worms were only scored if they appeared alive, healthy, and were not swimming (chambers were only scored if a majority of worms were alive and crawling). Worms were not scored within 2.5 cm to either side of the injection point.

To tally the location of each worm, pipettes were divided into 7 sections on each side of the 5 cm injection site. Each section was 3.5 cm long (the same length as a 1 mL volume indicated on the pipettes). The number of marker points in each section was then recorded. Data collection was validated with blinded replicates.

#### Other behavioral assays

A custom solenoid coil was constructed using a cardboard poster tube ∼60 cm in length and 10 cm diameter wrapped with 30 g wire spaced roughly 1 mm apart. For each assay, a Faraday cage consisting of aluminum foil and wire mesh was used inside the solenoid coil and a similarly constructed Faraday cage was used outside the coil in parallel. The Physics Toolbox Suite app for Android and iOS was used to measure the magnitude and direction of Earth’s magnetic field inside and outside the coil. The voltage from a Wanptek DC power supply unit (model number DPS305U) was then adjusted to reach an internal magnitude of 0 μT along the z axis inside of the solenoid coil. Experiments in the coil were run overnight as in other assays.

For parallel vs. perpendicular assays, chambers within our original Faraday cage were oriented with (parallel) or against (perpendicular) the direction of Earth’s magnetic field. Direction and magnitude were determined using Physics Toolbox Suite.

Temperature gradients were measured using an OMEGA HH506RA Multilogger Thermometer with T-type probes on multiple experimental days. The top and bottom of the Faraday cage was measured and found to be approximately 1°C warmer at the top versus the bottom, on average. To determine whether the upward movement observed in our assay was due to thermotaxis, we created a reverse gradient by placing the Faraday cage on a hot plate for the duration of the experiment, ensuring that the bottom of the assay was 1°C warmer than the top at the beginning and end of the experiment and monitored the temperature throughout. All experiments were performed at room temperature (ranging from ∼22°C to 25°C).

#### Calculations

Estimates of force due to gravity on *C. elegans* dauers were calculated based on known and experimentally determined physical parameters. The shape of a dauer larvae is roughly cylindrical with a radius and length of 10 μm and 450 μm, respectively.^49^

$$V=\pi 10\;\mu m^{2}\times 450\;\mu m=141.4\;\text{pL}$$

Therefore, the estimated volume of a dauer larvae is 141.4 pL. Estimates of *C. elegans* density vary based on experimental method^49^^,^^50^; assuming the maximum density of 1.09 g/mL (based on the empirically determined density of L2-L4 larvae), the force due to gravity on a dauers was approximated.

$$m=141.4\;pL\times 1.09\frac{g}{mL}≅154\;ng$$

$$F=154\;ng\times 9.8\frac{m}{s^{2}}=1.51\;nN$$

### Quantification and statistical analysis

Data were collected in Excel and analyzed using Rstudio. Worm positions across assays were summed and overall distributions were plotted for each strain or condition. Histograms depict the percentage of worms found at each location across all experiments. Variation between trials for Figure 2 can be found in the supplemental information (Figure S5). In some plots, the range shown along the x axis of the histograms varies to accommodate plots in which more worms were found at one location. Boxplots provide summaries of the distributions along with statistical significance.

In addition to horizontal controls, normal distributions with a mean of 0 were simulated to compare with vertical and other assays. These distributions were created either by keeping the standard deviation (Normal Distribution^a^) or the number of worms (Normal Distribution^b^) consistent with the combined set of vertical data for each condition. Keeping the standard deviation consistent led to a slightly reduced sample size in the simulated Normal Distribution^a^, as the distribution is wider than the fixed range of −7 to +7; however, these distributions represent 94% or more of the total vertical sample sizes in each case. Meanwhile, Normal Distribution^b^ has an identical sample size to each vertical condition at the expense of narrower standard deviations. Distributions were compared using Kruskal-Wallis followed by Dunn’s test and Bonferroni’s correction for multiple comparisons. In some instances, a Gravitaxis Index was calculated by subtracting the number of worms in the bottom of the assay from the number of worms in the top of the assay and dtividing by the total ($GI=\frac{\left(Top-Bottom\right)}{total}$). Statistical tests, *p* values, sample sizes, and replicates are detailed in the results and figure legends.

## Supplemental information

Document S1. Figures S1–S5 and supplemental references
