# Supplementary material for: Multimodal sensory inputs and mechanosensory components mediate C. elegans negative gravitaxis
Source: iScience. 2025 Oct 31;28(12):113923. doi: 10.1016/j.isci.2025.113923 (PMC12670890; doi:10.1016/j.isci.2025.113923)
Supplement: Document S1. Figures S1–S5 and supplemental references [file mmc1.pdf]

iScience, Volume *28*

Supplemental information

Multimodal sensory inputs and mechanosensory components mediate *C. elegans* negative gravitaxis

Caroline Ackley, Lindsey Washiashi, Neda Ziaei Kajbaf, Ruchira Krishnamurthy, Zhenxuan Sun, Vivian Duong, Kaylin Choe, Elijah Lane, Cricket Wood, Giulia Pellegrini, Eleanor Smith, Mark Sherwin, Pradeep Joshi, and Joel H. Rothman

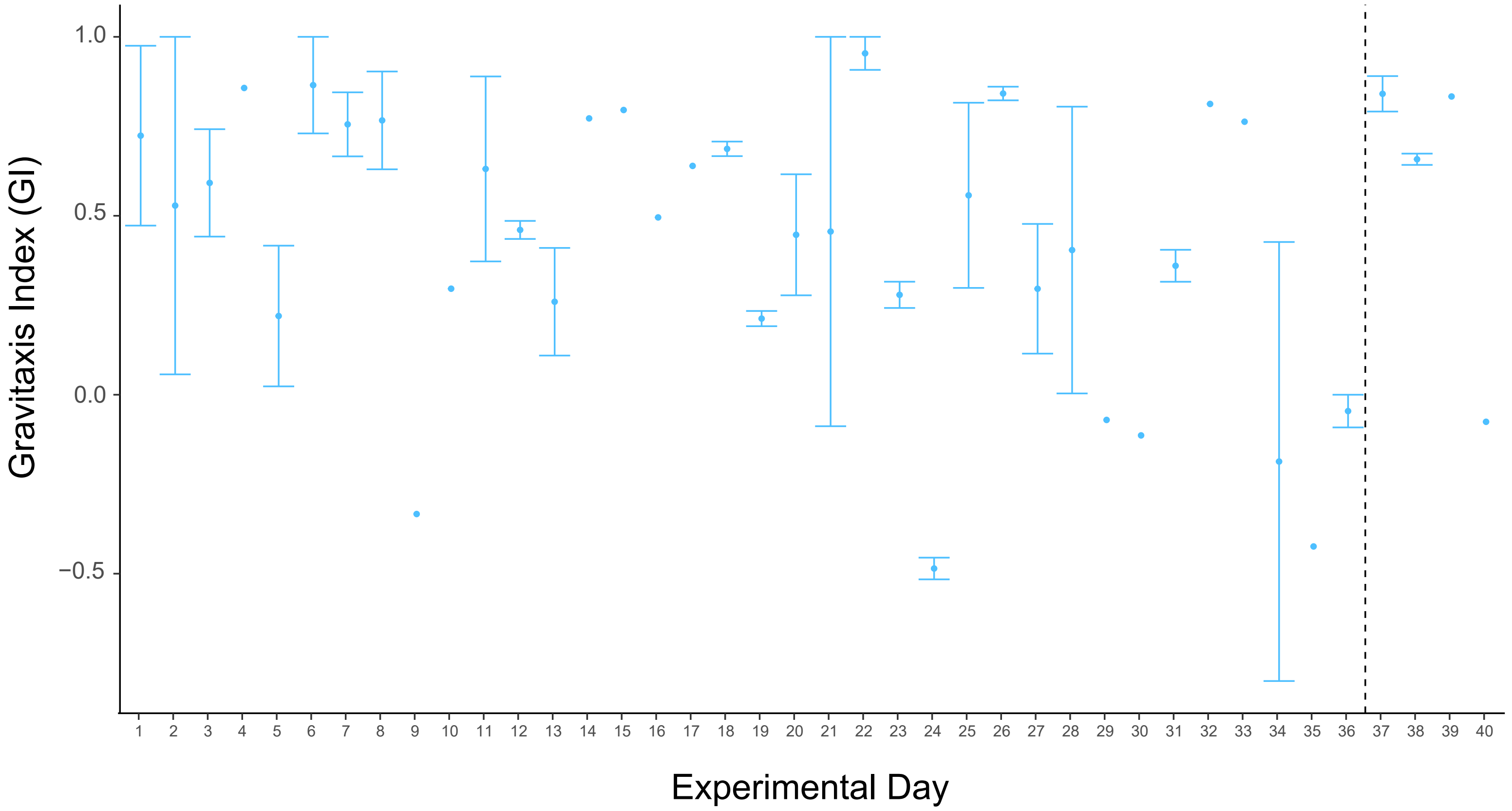

Figure S1. Faraday cage integrity influences gravitactic behavior, Related to Figure 2
Plot of Gravitaxis Index (GI) – calculated as the sum of worms in the top half of the chamber (segments +1 to +7) minus worms in the bottom half (-1 to -7) divided by the total – for all chambers across Different experimental days. Calculated for N2 dauers only. Dashed line indicates reinforcement of the Faraday cage.

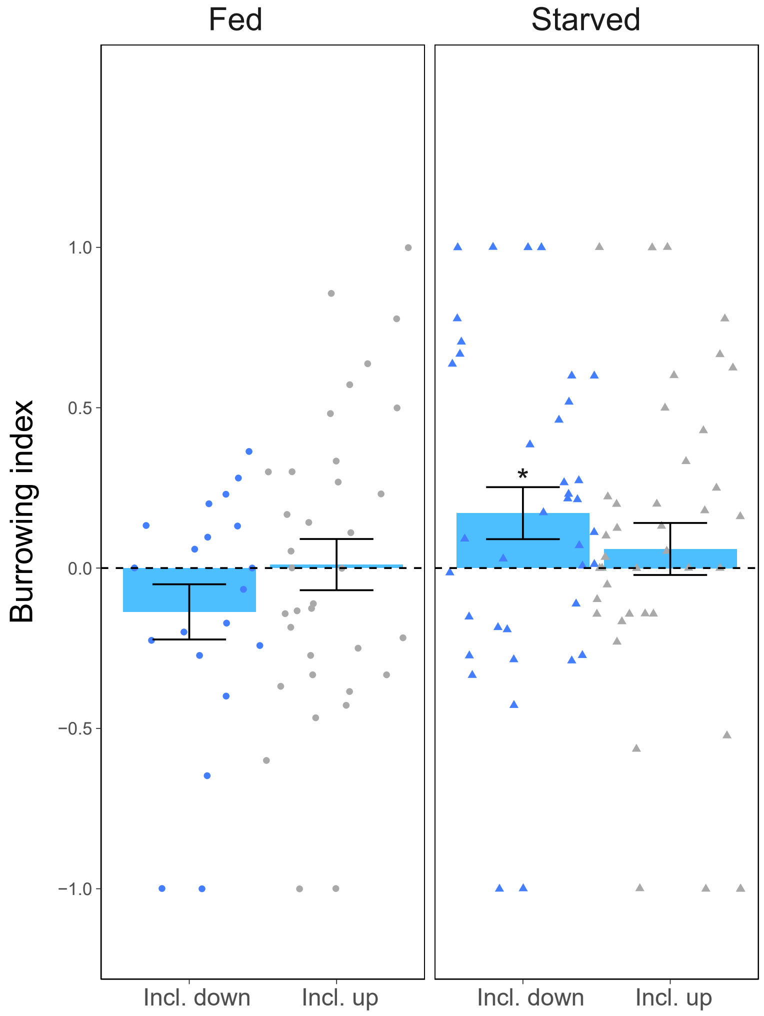

Figure S2. A negative gravitactic trend is also observed in *Landler et al., 2018*[1], Related to Figure 5
Data reprinted and new statistics run with permission from the authors. In the original paper, burrowing behavior was measured in an artificial magnetic field similar to the solenoid coil experiments presented in this study. The burrowing index for each trial (equivalent to the gravitaxis index) have been plotted with circles representing fed adult N2 worms, triangles representing starved worms, and blue and grey symbols representing worms exposed to positive and negative field inclinations, respectively. A two-sided t-test was performed by the authors of this study and annotated accordingly (* $p < 0.05$).

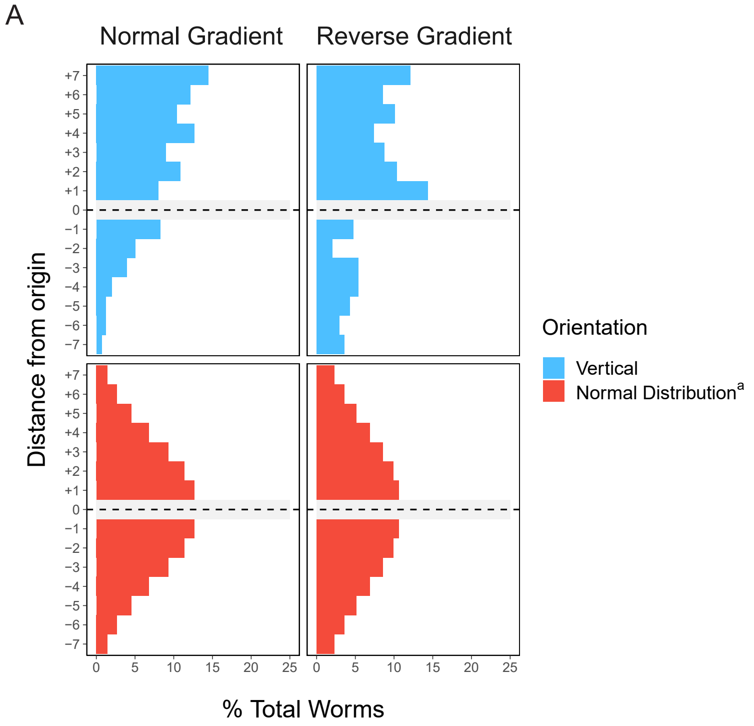

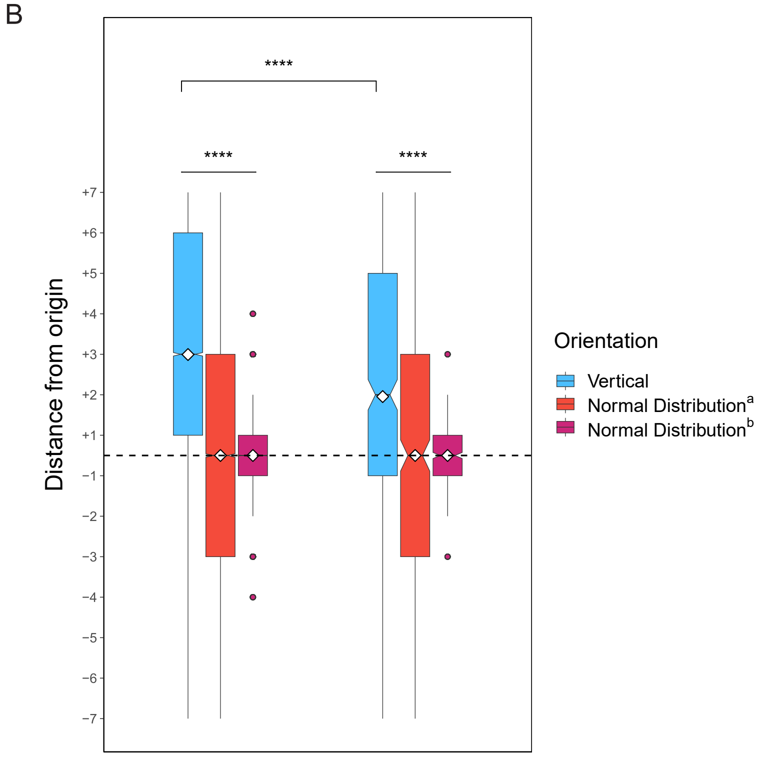

Figure S3. Gravitaxis behavior occurs independently from thermotaxis along shallow temperature gradients, Related to Figure 6

(A) Histograms depicting horizontal and vertical movement of N2 dauers along normal and reverse temperature gradients. **(B)** Boxplots summarizing data shown in A. Reverse gradient n = 445 worms over 5 trials. * $p < 0.05$, ** $p < 0.01$, *** $p < 0.001$, **** $p < 0.0001$; n.s. is not significant using Kruskall-Wallis followed by Dunn's test with Bonferroni correction. Notches on boxplots represent 95% confidence intervals; mean values are indicated with a diamond.

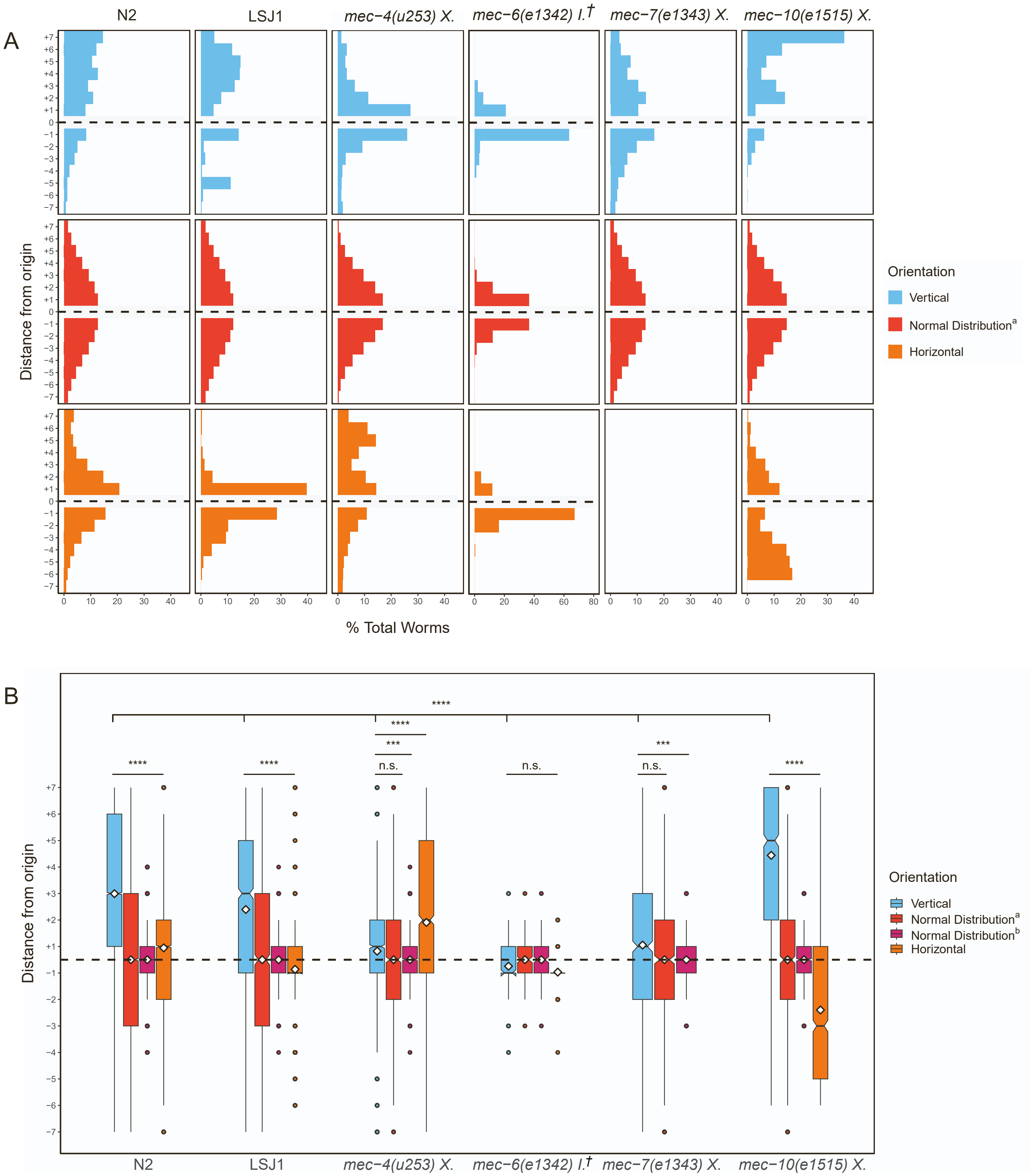

Figure S4. Gravitaxis behavior in additional strains, Related to Figure 7
(A) Histograms depicting horizontal and vertical movement of N2 and LSJ1 worms as well as additional mechanosensory deficient dauers. *mec-4(u253)* and *mec-6(e1342)* dauers were found to be sluggish, resulting in little taxis in either direction. †*mec-6* dauers accumulated at the origin of the assay; the x-axis was altered in plot to allow for visual comparison. Additional alleles of mec-7 and mec-10 recapitulate the findings shown in results.**(B)** Boxplots summarizing data shown in A. LSJ1 vertical n = 1,666 worms over 7 trials; horizontal n = 2,510 worms over 7 trials. *mec-4(u253)* vertical n = 2,075 worms over 7 trials; horizontal n = 2,130 worms over 4 trials. *mec-6(e1342)* vertical n = 195 worms over 6 trials; horizontal n = 277 worms over 4 trials. *mec-7(e1343)* vertical = 949 worms over 8 trials. mec-10(e1515) vertical n = 1,244 worms over 4 trials; horizontal n = 1,334 worms over 2 trials. * p < 0.05, ** p < 0.01, *** p <0.001 , **** p < 0.0001; n.s. is not significant using Kruskall-Wallis followed by Dunn's test with Bonferroni correction. Notches on boxplots represent 95% confidence intervals; mean values are indicated with a diamond.

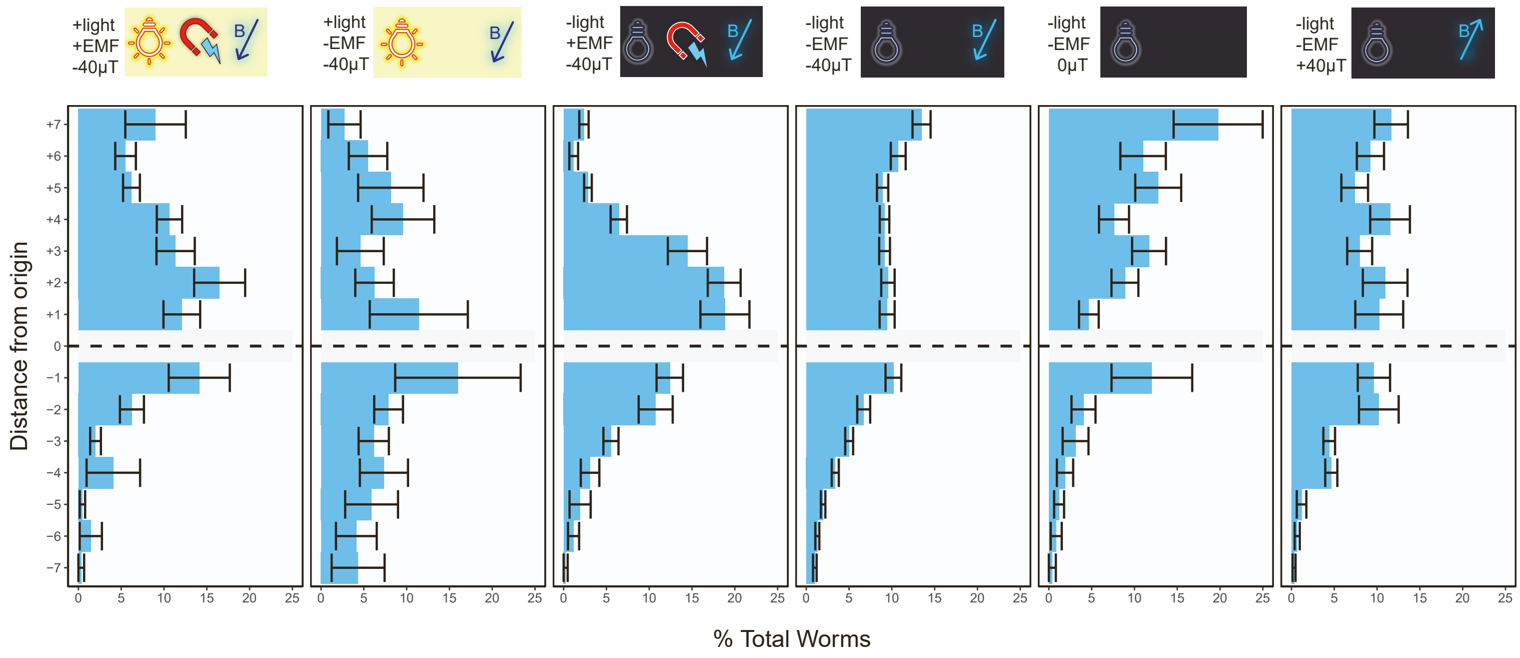

n	2222	542	4358	35816	696	1112
trials	10	6	12	148	9	9

Figure S5. Worm distribution variation between trials, Related to STAR Methods
Histograms depicting vertical movement of N2 worms as shown in Figure 2. SEM is plotted at each vertical location to show variation in worm location between trials. Sample sizes and trial numbers are listed in the table below.

Supplemental References

[S1] L. Landler, S. Nimpf, T. Hochstoeger, G. C. Nordmann, A. Papadaki-Anastasopoulou, and D. A. Keays, “Comment on ‘Magnetosensitive neurons...,’” *eLife*, vol. 7, 2018, doi: 10.7554/elife.30187.001.
